# Microglia in the degenerating brain are capable of phagocytosis of beads and of apoptotic cells, but do not efficiently remove PrP^Sc^, even upon LPS stimulation

**DOI:** 10.1002/glia.21070

**Published:** 2010-09-27

**Authors:** Martina M Hughes, Robert H Field, V Hugh Perry, Carol L Murray, Colm Cunningham

**Affiliations:** 1 Trinity College Institute of Neuroscience and School of Biochemistry and Immunology, Trinity College DublinDublin 2, Republic of Ireland; 2 School of Biological Sciences, University of SouthamptonSouthampton, United Kingdom

**Keywords:** amyloid, microglia, priming, phagocytosis, activation, prion disease, apoptosis, Alzheimer's disease

## Abstract

Despite the phagocytic machinery available to microglia the aberrant amyloid proteins produced during Alzheimer's and prion disease, amyloid-β and PrP^Sc^, are inefficiently cleared. We have shown that microglia in the ME7 model of prion disease show morphological evidence of activation, synthesize low levels of pro-inflammatory cytokines and are primed to produce exaggerated responses to subsequent inflammatory challenges. Whether these microglia engage in significant phagocytic activity in the disease per se, or upon subsequent inflammatory challenge is not clear. In the present study we show transcriptional activation of a large number of scavenger receptors (SRs), matrix metalloproteinases (MMPs), oxidative enzymes, and cathepsins in ME7 animals. Hippocampally-injected inert latex beads (6 μm) are efficiently phagocytosed by microglia of ME7 prion-diseased animals, but not by microglia in normal animals. Stimulation of ME7 animals with systemic bacterial endotoxin (lipopolysaccharide, LPS) induced further increases in SR-A2, MMP3, and urokinase plasminogen activator receptor (uPAR) but decreased, or did not alter, transcription of most phagocytosis-related genes examined and did not enhance clearance of deposited PrP^Sc^. Furthermore, intracerebral injection with LPS (0.5 μg) induced marked microglial production of IL-1β, robust cellular infiltration and marked apoptosis but also did not induce further clearance of PrP^Sc^. These data indicate that microglia in the prion-diseased brain are capable of phagocytosis per se, but show limited efficacy in removing PrP^Sc^ even upon marked escalation of CNS inflammation. Furthermore, microglia/macrophages remain IL-1β-negative during phagocytosis of apoptotic cells. The data demonstrate that phagocytic activity and pro-inflammatory microglial phenotype do not necessarily correlate. © 2010 Wiley-Liss, Inc.

## INTRODUCTION

Microglia represent the largest population of phagocytes in the CNS and have a principal role in immune defense and inflammatory responses in the CNS (Minghetti et al., 2005; Napoli and Neumann, 2009; Ransohoff and Perry, 2009). In the normal healthy brain, microglia exhibit a highly ramified “resting” morphology (Raivich et al., 1999; Streit et al., 1999) although they are now known to be active, and continually scanning their immediate vicinity for pathogens or disturbances of homeostasis (Nimmerjahn et al., 2005).

Upon exposure to pathogenic or other stimulatory molecules, microglial surface receptors including Toll-like receptors (TLRs), complement receptors (CRs), Fc receptors, and scavenger receptors (SRs) recognize particular moieties or macromolecules, allowing the binding and engulfment of these molecules (see Lucin and Wyss-Coray, 2009 for review). One typical consequence of this is the release of cytokines such as tumor necrosis factor alpha (TNF-α), interleukin-1β (IL-1β), and interleukin-6 (IL-6), (Napoli and Neumann, 2009). Microglia also become activated, as evidenced by their more condensed morphology, upon encountering apoptotic and neuronal debris but in this case appear to adopt an antiinflammatory phenotype characterized by the expression of molecules such as Triggering receptor expressed on myeloid cells-2 (TREM2), Transforming Growth Factor- Beta (TGF-β1), and Interleukin 10 (Cunningham et al., 2002; De Simone et al., 2003, 2004; Takahashi et al., 2005).

AD and prion diseases show many similarities in that they are typified by the accumulation of compact amyloid protein, marked neuronal degeneration, and significant morphologically activated microglia and astrocytosis (DeArmond, 1993; Soto et al., 2003). The failure to clear the abundant amyloid deposition in these diseases, despite apparent robust morphological evidence of microglial and astroglial activation, suggests that phagocytosis is somehow deficient in these diseases. It has been proposed that microglia become more activated as they become more phagocytic and that this occurs in a graded, linear, and reversible fashion (Raivich et al., 1999; Streit et al., 1999). According to this scheme morphology is a key indicator of “activation state” or phenotype. However, we have shown that morphology alone is not a good predictor of inflammatory phenotype of microglia and that cells primed by prior pathology can make significant changes in their secretory profile without any apparent change in morphology (Cunningham et al., 2005). Microglia in the ME7 model of prion disease, despite evidence of robust morphological activation, synthesize very low levels of proinflammatory cytokines but elevated TGF-β1, inducible cyclooxygenase (COX2), and prostaglandin E2 (PGE2) (Cunningham et al., 2002; Walsh et al., 2000, 2001). This profile is characteristic of macrophages following the phagocytosis of apoptotic cells (Fadok et al., 1998; Savill et al., 2002) or of microglia during phagocytosis of phosphatidyl serine (PS)-expressing liposomes to mimic apoptotic cells (De Simone et al., 2003, 2004). Collectively these data suggest that microglia may be active in phagocytosis of neuronal debris during chronic neurodegeneration but perhaps are not efficient phagocytes with respect to PrP^Sc^.

In this study we sought to demonstrate that the basic phagocytic machinery is functional in the ME7 model of prion disease by examining the transcription of a wide variety of phagocytosis and extracellular proteolysis-associated molecules: the scavenger receptors A2 (SRA2), B (CD36), receptor for glycation end products (RAGE), and macrosialin (CD68); the matrix metalloproteinases MMP 3, 9, and 12; lysosomal cathepsins D, S, Z, and H; NADPH oxidase subunits p22 phox, p91 phox; myeloperoxidase (MPO); phagocytic receptors, TREM2 and CD200 receptor and the serine proteases tissue plasminogen activator (tPA) and urokinase PA (uPA). Since many of these transcripts are not myeloid lineage-specific we also functionally assessed phagocytosis in microglia. Microglia in the naïve brain are only weakly phagocytic with respect to inert 1 μm fluospheres (Carare et al., 2008) but internalization of small particles may also be achieved by pinocytosis (Falcone et al., 2006). To examine uptake by phagocytosis while excluding pinocytosis/macropinocytosis, which typically accommodates from 0.5 to 2.5 μm (Swanson and Watts 1995), we have assessed the engulfment of inert 6-μm latex beads injected directly into the hippocampus of normal and prion-diseased animals.

In addition, marked reduction in amyloid-β following intra-hippocampal LPS injections has been demonstrated in transgenic mouse models of Alzheimer disease (DiCarlo et al., 2001; Herber et al., 2004) and focal clearance of amyloid-β has been reported in small ischemic lesions in Alzheimer's diseased-brain (Akiyama and McGeer, 2004), suggesting that further microglial activation induces further amyloid clearance. Thus, we have examined whether primed microglia of the prion-diseased brain can affect more efficient clearance of PrP^Sc^ upon further activation by systemic or central LPS challenges. We examined further changes in the transcriptional profile of the disease-associated microglia, assessed for enhanced clearance of PrP^Sc^ and characterized the inflammatory and apoptotic consequences of further activation of the degenerating brain.

## MATERIALS AND METHODS

### Animals, Treatments, and Tissue Preparation

Female C57BL/6 mice (Harlan Olac, UK), 8- to 10-weeks old, were housed under standard conditions in groups of three to five, with food and water *ad libitum*. Animals for surgery were intraperitoneally (i.p.) anaesthetized with Avertin (Sigma, Poole, UK) and positioned in a stereotaxic frame. The mice were injected into the dorsal hippocampus on both sides with either a 10% w/v ME7-infected C57BL/6 brain homogenate (ME7 animals) or a 10% w/v normal brain homogenate (NBH animals) made in phosphate buffer saline (PBS). At 18-weeks postinoculation ME7 animals or NBH animals (*n* = 6) were further challenged with 1 μL of a suspension of naked latex beads (Molecular Probes, Eugene, Oregon, 6 μm, 5 × 10^5^ μL) and perfused 4 h later. The 4-h time point was based on the time course of latex bead phagocytosis by macrophages *in vitro* (Nick Platt, personal communication) and 18-weeks postinoculation with ME7 is the disease stage at which we have described microglial priming with subsequent LPS induced switching of phenotype (Cunningham et al., 2005). Further ME7 animals (*n* = 6) received an intracerebral challenge with LPS (0.5 μg) and were sacrificed at either 24 or 72 h after treatment, or an intraperitoneal injection with LPS (500 μg kg^−1^, *n* = 3), while NBH animals (*n* = 3) were injected i.c. with LPS and sacrificed at 24-h posttreatment or injected i.p. with saline and sacrificed at 72 h (*n* = 3). Sham surgeries were carried out to provide ME7 controls (*n* = 3) since intra-cerebral insertion of even a microcapillary could constitute a significant inflammatory stimulus in the primed ME7 brain. Following formalin perfusion the brains were removed and postfixed in 10% formalin solution. For mRNA quantification studies, a second group of mice (*n* = 10 ME7, *n* = 8 NBH) were challenged intraperitoneally with either LPS (500 μg kg^−1^), previously shown by us (Cunningham et al., 2005) to significantly alter microglial IL-1β expression, or saline and were terminally anaesthetized and perfused with heparinized saline 3-h postinjection. The hippocampus from each animal was quickly removed, and immediately frozen in liquid nitrogen, before being stored in the −80°C freezer until required for RNA extraction.

### Tissue Processing and Immunohistochemistry

Perfused, formalin-fixed brains were dehydrated and paraffin wax-embedded and sections of 10 μm thickness were cut using a microtome (Leica RM2235). Cut sections were floated on a water bath heated to 50°C and collected onto positively charged SuperFrost®Plus Microscope Slides (VWR International). Fresh frozen sections were cut on a cryostat (Leica), collected on gelatin-coated slides and air-dried overnight before storage at −20°C.

### CD68 and Latex Beads

Fresh frozen sections were stained for the endosomal/lysosomal marker CD68 using the FA11 antibody (Serotec, Oxford, UK) by the ABC method using diaminobenzidine as chromagen, as previously described (Cunningham et al., 2003). These slides were not dehydrated before mounting since this damages the integrity of the latex beads, and slides were instead mounted using aqueous mounting medium (Citifluor, Leicester, UK). Slides were examined by light microscopy. The number of latex beads completely surrounded by CD68-positive staining was counted in the ipsilateral hippocampus of injected NBH- and ME7 animals. All beads located in the meninges/pial membranes were omitted from counting and likewise all beads forming clusters of more than 20 beads were also omitted (since tight clusters might prevent access of microglial cells to individual beads). Since the numbers of beads present in the parenchyma and available (i.e., not clustered) varied from animal to animal the ratio of CD68-positive beads to CD68-negative beads was calculated for each animal. Thus, all beads that were dispersed or clustered in small groups (<20) within the parenchyma were counted in three sections adjacent to the injection site and totaled. The total number of beads phagocytosed was divided by the total not phagocytosed to calculate a ratio for each animal and these ratios were averaged for three animals in each treatment group. These data are thus presented as ratios of beads phagocytosed to beads not phagocytosed.

### Immunohistochemistry in Paraffin-Embedded Tissue

Formalin-fixed, paraffin-embedded sections were assessed for expression of IL-1β, PrP^Sc^, IBA-1, myeloperoxidase, and fragmented DNA (TUNEL) using antibodies against the relevant antigens. For IL-1β and IBA-1, sections were dewaxed and rehydrated, quenched for 20 min with 1% hydrogen peroxide (H_2_O_2_) in absolute methanol to eliminate nonspecific peroxidase activity and microwaved twice for 5 min on high power in citrate buffer pH 6, with 5-min cooling between each incubation. Slides for IL-1β were washed in PBS, blocked for 40 min with 20% normal goat serum and incubated at 4°C overnight in rabbit polyclonal anti-IL-1β primary antibody diluted 1:50 in PBS (Peprotech, London UK) while those for IBA-1 were washed in PBS, blocked in 10% normal rabbit serum and incubated overnight at 4°C in anti-IBA-1 primary antibody diluted 1/200 in PBS-Tween (Abcam, Cambridge, UK). After washing and 40 min-incubation with biotinylated goat antirabbit IgG or rabbit antigoat IgG (Vector, LabKem, Dublin), avidin-biotin-peroxidase complex (ABC) solution was dropped on each section and left for 40 min to incubate. The DAB reaction (300 mL 0.1 M phosphate buffer, 125 μL H_2_O_2_) was monitored under the microscope to ensure optimal incubation time. The sections were then counter stained in Harris' Haematoxylin (VWR, Dublin) and dehydrated before coverslipping with DPX.

The sections for PrP^Sc^ staining were rehydrated and underwent hydrated autoclaving at 121°C for 20 min and Proteinase K (50 μg mL^−1^) digestion for 30 min to destroy normal PrP^c^. The sections were briefly washed in PBS before being placed in 90% formic acid for 5 min. Sections were then quenched in 1% H_2_O_2_ in absolute methanol and washed in PBS before blocking in 10% horse serum. Primary antibody 6D11 (Santa Cruz Biotechnology, Santa Cruz, CA) was diluted 1 in 100 in PBS was left to incubate overnight at 4°C. Biotinylated horse anti-mouse IgG (Vector) was pipetted onto the sections and incubated for 40 min, followed by incubation with ABC and the DAB reaction as described above.

Sections for MPO labeling were assessed using an anti-MPO serum, used undiluted (Abcam, Cambridge, UK), following the standard protocol for quenching, washing, and citrate pretreatment. The sections were also washed once with PBS-Triton (1%) before blocking and overnight primary antibody incubation. TUNEL staining was performed using (Promega, Southampton, UK) Colorimetric Dead End kit as per manufacturers instructions.

Immunohistochemically labeled sections were photographed using a Olympus DP25 camera (Mason, Dublin) counted on a Leica DM3000 microscope (Laboratory Instruments and Supplies, Dublin) and captured using cell A imaging software. Density analysis was performed using ImageJ software using the “measure” function applied to rectangles of equal area in the hippocampus of ME7 animals. In the case of intra-peritoneal LPS injections the PrP^Sc^ densities of the stratum radiatum, stratum oriens, and corpus callosum were measured in animals 72 h after intraperitoneal challenge with saline or LPS (500 μg kg^−1^). The transmittance in the corpus callosum was chosen as an internal standard for its high transmittance (low density) and measures for the strata oriens and radiatum were subtracted from this in order to standardize for variability in staining from one section to another (i.e., transmittance_CC_ − transmittance_SR_ = density_SR_). In the case of intracerebral LPS challenges, similar area rectangles were taken in the stratum radiatum and oriens, proximal to the intra-hippocampal LPS injection tract and in the same regions on the contralateral side as well as in SHAM-operated ME7 animals.

### Double Labeling and Confocal Microscopy

Double labeling experiments were performed using the primary antibodies as described above and using Alexafluor secondary reagents as follows: Goat anti-rabbit 488 (IL-1β), Donkey anti-mouse 568 (NeuN), Donkey anti-goat 594 (IBA-1), and goat anti-rabbit 647 (MPO) (Invitrogen, Biosciences, Dublin). The promega fluorometric Dead end kit was used in place of the colorimetric one, thus incorporating fluorescein-labeled nucleotides (into fragmented DNA in the apoptotic cell. Where incompatibilities arose between blocking sera and secondary antibodies required, 5% BSA was used in the place of sera from particular species. Sections were counterstained for 5 min using Hoescht 33258 (1 μg mL^−1^) before rinsing in PBS and mounting in ProLong Gold (Invitrogen, Biosciences, Dublin). Double-labeled sections were visualized and captured on an Olympus FV1000 confocal microscope using sequential excitation at 404, 488, and 543 and using 40× (numerical aperture 0.9) and 60× oil (numerical aperture 1.35) objectives. Images shown are single sections of <1 μm extracted from Z-stacks of 10-μm sections.

### RNA Isolation, cDNA Synthesis, and Quantitative PCR

Total RNA was isolated using the RNeasy Plus Mini method (Qiagen) as per the manufacturers instructions. To ensure complete DNA elimination from the column-bound RNA, an on column DNase step was performed. The RNA yield and quality of each sample was quantified based on Optical Density (OD) using the NanoDrop©ND-1000 UV-Vis spectrophotometer (Thermo Fisher Scientific). cDNA synthesis was carried out using a High Capacity cDNA Reverse Transcriptase Kit (Applied Biosystems). An RNA master mix was made up as follows: 2 μL 10× RT Buffer, 0.8 μL 25× dNTP mix, 100 mM; 2 μL 10× RT random primers, 1 μL MultiScribe™ Reverse Transcriptase, 4.2 μL RNase-free water and 10 μL RNA was added (200 ng/10 μL) and the reaction completed in the thermocycler (Peltier Thermal Cycler PTC-200) at 25°C for 10 min; 37°C for 120 min and 85°C for 5 min. cDNA was stored at −20°C until use in RT PCR to analyze expression of genes of interest (see Supp. Info. Table 1). All primer and probe sets were designed using Applied Biosystems Primer Express software and amplified a single sequence of the correct amplicon size, as verified by SDS-PAGE. Where no probe sequence is shown, the DNA binding dye SYBR green was used in its place. Samples for RT-PCR were run in duplicate and contained 12.5 μL Taqman®Universal PCR Master Mix (Applied Biosystems), 0.5 μL of each of the forward primer (10 μM), reverse primer (10 μM) and probe (10 μM), and 10 μL RNase-free water. Dual-labeled probes used the 5′ reporter dye FAM and the 3′ quencher TAMRA. All PCR was carried out in a real-time PCR thermocycler (Applied Biosystems 7300 Real-time PCR System) under the cycling conditions: 50°C for 2 min, 95°C for 10 min, 95°C for 15 s, and 60°C for 1 min. Quantification was done exploiting the relative quantitation method, using LPS-injected mouse brain as a standard expressing all genes of interest and fourfold serial dilutions of this cDNA to construct a linear standard curve relating cycle threshold (*C*_T_) values to relative concentrations. This method has been described in detail elsewhere (Cunningham et al., 2005). Gene expression data were normalized to the housekeeping gene glyceraldehyde-3-phosphate dehydrogenase (GAPDH), which acts as an endogenous control for quantification assays.

### Statistical Analysis

All data were analyzed using Graph Pad Prism software. Students *t*-tests were performed to compare gene expression changes in ME7 animals versus NBH animals and to compare PrP^Sc^ densities after i.p. challenges. PrP^Sc^ densities after i.c. challenge were assessed by one-way ANOVA and gene expression data in four treatment groups were also analyzed by one-way ANOVA followed by selected pairwise comparisons using Bonferroni post-hoc tests. Data were considered significant when *P* < 0.05 and were presented as group means ± S.E.M.

## RESULTS

### Upregulation of Phagocytosis-Related Genes in ME7 Versus NBH Animals

At 18-weeks postinoculation with ME7 there was marked upregulation in a large number of genes associated with phagocytosis and extracellular and intracellular proteolysis. Fold increases with respect to levels of expression of these transcripts in NBH animals are shown in Table 1.

**TABLE 1:** Expression of mRNA for Phagocytic, Proteolytic, Oxidative and Microglial Gene Products

Transcript	Fold increase (ME7:NBH)	*t* test *P* value
Scavenger receptors		
SRA2	9.3	0.0038
SRB (CD36)	2.2	0.0009
CD68	13.4	<0.0001
RAGE	3.9	<0.0001
Cathepsins		
Z	8.2	<0.0001
S	11.7	<0.0001
D	14.6	<0.0001
H	7.6	<0.0001
Extracellular proteases		
MMP3	24	0.0009
MMP9	1.3	0.0024
MMP12	31.1	<0.0001
tPA	3	0.0052
uPA	20.2	<0.0001
Oxidative burst		
p22phox	13.7	<0.0001
p91phox	50.6	<0.0001
Myeloperoxidase	1.1	0.7505
Other microglial		
TREM2	12.9	<0.0001
CD200R	6.7	<0.0001
uPAR	13.4	<0.0001
PBZR	5.9	<0.0001

The scavenger receptors SRA2, CD68, and SRB (CD36) are restricted to macrophage lineage cells and some endothelial populations, are generally expressed at low levels in the normal brain and are upregulated in microglia upon exposure to various stimuli (see Husemann et al., 2002 for review). Receptor for advanced glycation end-products (RAGE), while certainly expressed by microglia, may also show a broader distribution (Lue et al., 2005). Here, CD68 and SRA-2 transcripts were upregulated by ∼13- and 9-fold, respectively, while CD36 and RAGE showed 2- and 4-fold inductions, respectively. These differences were all statistically significant as assessed by students *t* test (*P* = 0.0038 for SRA2, *P* = 0.0009 for CD36, and *P* < 0.0001 for other scavenger receptors).

Respiratory burst-associated genes such as the p91phox and p22phox subunits of NADPH oxidase are predominantly expressed by professional phagocytes such as macrophages/microglia and neutrophils, but NADPH oxidase is now recognized to be active in neurons and perhaps astrocytes (see Sun et al., 2007 for review). These transcripts were robustly increased in expression in prion disease. Approximate fold increases for p91phox and p22phox subunits of NADPH oxidase were 50- and 14-fold, respectively and these were statistically different to NBH animals' expression levels (*P* < 0.0001). Conversely myeloperoxidase did not show any evidence of induction (*P* = 0.75).

Cathepsins are widely distributed and can be expressed by multiple CNS cell types (Nakanishi, 2003; Nixon et al., 2000). Those examined in this study (Z, S, D, H) have been shown to be expressed by microglia and indeed cathepsins H, S, and Z show some selectivity for myeloid lineage cells (Akahoshi et al., 2007; Kominami et al., 1985; Wendt et al., 2008) though the former two are also elevated in astrocytomas (Flannery et al., 2003; Kominami et al., 1985). Cathepsin D is more commonly described in neurons (Nakanishi, 2003; Puyal et al., 2009). All cathepsins studied here showed increases of ∼8- to 15-fold (*P* < 0.0001 for all four cathepsins).

The extracellular proteases uPA, tPA, and MMPs 3, 9, and 12 are also expressed by multiple cell types and here showed more variable induction: MMP3 was significantly increased from negligible levels in NBH animals, such that fold differences may be exaggerated (*P* = 0.0009). MMP12 showed ∼30-fold increases compared with NBH animals (*P* < 0.0001) while MMP9, though statistically significant (*P* = 0.0024), showed only a 1.3-fold increase. The expression of tPA was increased by ∼3-fold (*P* = 0.0052), while that of uPA was increased 20-fold (*P* < 0.0001).

TREM2, which is myeloid restricted and thought to be important in the noninflammatory phagocytosis of apoptotic cells (Takahashi et al., 2005), was induced ∼13 fold in ME7 animals with respect to NBH animals (*P* < 0.0001). CD200R, which is also myeloid restricted and thought to maintain a predominantly antiinflammatory phenotype in microglial cells (Masocha, 2009), was elevated ∼7 fold in ME7 with respect to NBH (*P* < 0.0001). The urokinase plasminogen activator receptor, uPAR, recently shown to be expressed by microglia in diverse pathologies (Cunningham et al., 2009b), was elevated 13 fold (*P* < 0.0001) and the peripheral benzodiazepine receptor (PBZR), previously used to identify activated microglia in positron emission tomography (PET) imaging studies (Cagnin et al. 2001) was elevated 6 fold (*P* < 0.0001).

### Phagocytosis of 6-μm Latex Beads

**Fig 1:**
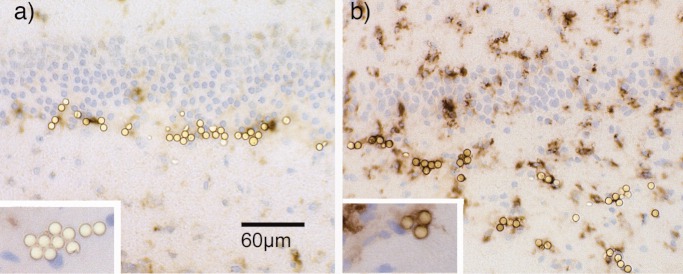
Phagocytosis of inert latex beads. Latex beads (6 μm) were injected intra-hippocampally into (a) NBH- and (b) ME7 animals at 18-weeks postinoculation and their engulfment by CD68-positive microglial cells assessed by immunohistochemistry. Slides were viewed by bright field microscopy at ×20 magnification. Beads were designated phagocytosed if CD68 labeling was apposed to the entire circumference of the bead (b, inset). There were limited interactions between beads and CD68-positive cells in NBH animals (a, inset) compared with robust engulfment observed in ME7 animals. Scale bar = 60 μm.

The data in Table 1 are suggestive of an upregulation of phagocytic and proteolytic machinery, but since these transcripts are not all exclusively microglial and also do not demonstrate phagocytosis per se, we wished to independently show that microglial phagocytosis is functional in the ME7 animals. Latex beads, 6 μm in diameter, cannot be taken up by macropinocytosis and hence are only taken up by phagocytosis (Hewlett et al., 1994). Their phagocytosis here is assessed by association with the myeloid restricted endosomal/lysosomal marker CD68, indicating complete engulfment by the endosome/lysosome. When injected into NBH animals the beads show limited association with CD68-labeled microglial cells (Fig. 1a). Conversely, in ME7 animals clear association of latex beads with CD68-positive microglia is apparent in Fig. 1b. In all ME7 animals injected the beads were associated with CD68-positive cells and were apposed by CD68 along the entire circumference of the bead, indicative of complete engulfment. When calculated as a ratio of phagocytosed to nonphagocytosed beads, microglia in ME7 animals engulfed available beads at a ratio of 3.58 ± 0.41 : 1 while microglia in NBH animals engulfed beads at a ratio: 0.19 ± 0.01 : 1. These ratios are statistically significantly different by *t* test (*P* = 0.0007) and this indicates a significant engagement of the phagocytic machinery by inert latex beads in ME7 animals but very limited phagocytic activity in NBH animals. More simply, ME7 animals are more phagocytically active, engulfing ∼78% of available beads while NBH animals engulf ∼19%.

### Systemic Challenge with LPS Induces Further Transcriptional Changes in ME7 Prion-Diseased Mice with Respect to NBH Controls

Collectively the above data demonstrate that microglia in the prion-diseased brain show robust phagocytic capacity, however PrP^Sc^ persists in the tissue. We wished to examine the degree to which CNS transcriptional profiles and PrP^Sc^ clearance might be altered by the induction of systemic inflammation in NBH- and ME7 animals. NBH- and ME7 animals were challenged i.p. at 18 weeks with either LPS (500 μg kg^−1^) or saline. Relative mRNA expression was assessed for all genes of interest 3 h after intraperitoneal challenge with LPS. Many genes were not significantly altered by systemic treatment with LPS (in either ME7- or NBH- animals). These genes included MMP9, MMP12, tPA, MPO, TREM2, CD200R, PBZR, and RAGE (data not shown). CD68, p91 and p22 showed slight decreases in mRNA upon subsequent LPS challenge but these changes were not significant.

**Fig 2:**
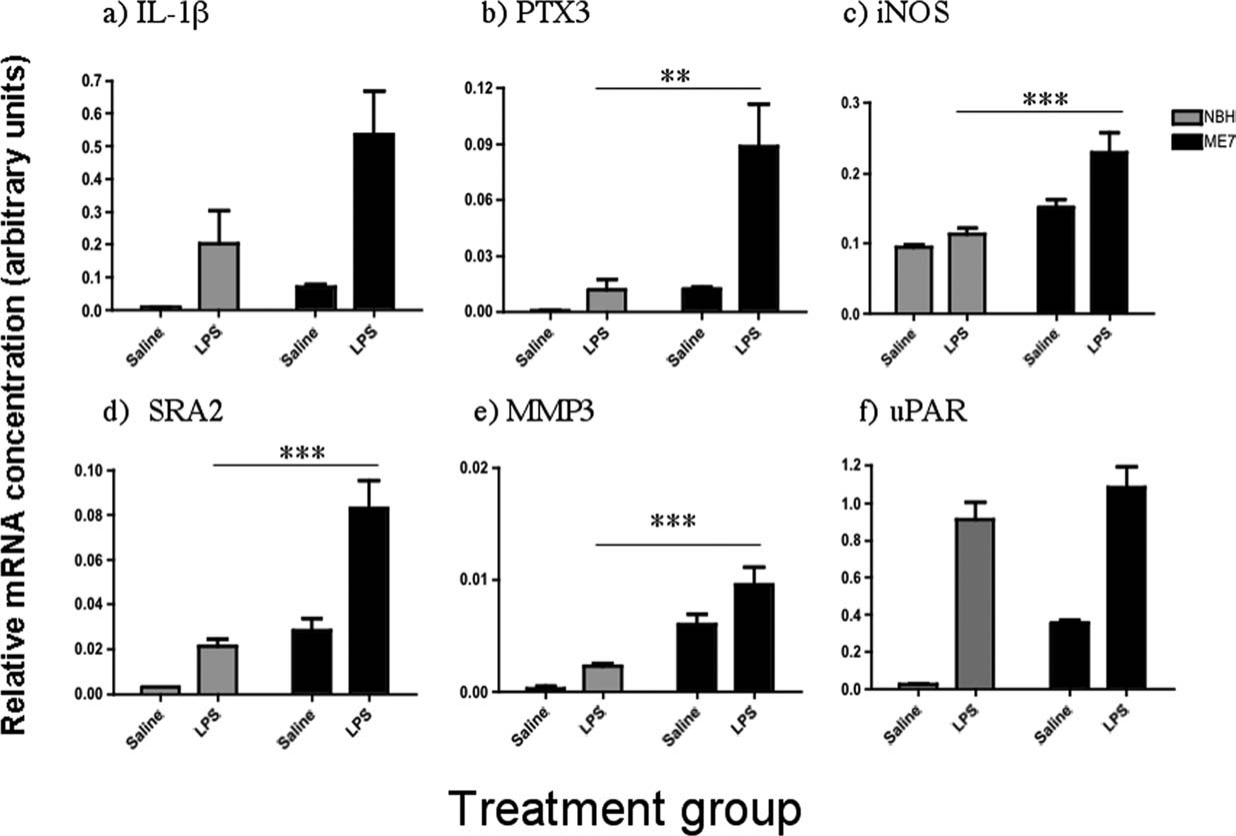
Further induction of inflammatory genes by LPS. Systemic challenge with LPS (500 μg kg^−1^) induced a number of transcripts in the hippocampus of ME7 animals to a greater extent than in NBH animals. (a) IL-1β, (b) PTX3, (c) iNOS, (d) SRA2, (e) MMP3, and (f) uPAR. Significant differences between ME7+LPS and NBH+LPS, as shown by one way ANOVA followed by Bonferroni post-hoc tests, are shown by **P < 0.01, ***P < 0.001. *n* = 3 for NBH+saline and *n* = 5 for all other groups.

A number of transcripts showed increased expression after LPS stimulation. Confirmation of further phenotypic change of the primed microglial state is provided by the increased expression of IL-1β, PTX3, iNOS and is shown (Fig. 2a–c respectively).

Following systemic challenge with LPS, IL-1β mRNA showed an increase in NBH animals compared with saline treatment, however, the same dose of LPS produced a much greater increase in IL-1β expression in ME7 animals (Fig. 2a). This difference was not statistically significant due to the presence of one high value in NBH+LPS. PTX3 was also elevated in ME7+LPS mice compared with all other treatment groups (*P* < 0.01, Fig. 2b). Similarly iNOS mRNA was also significantly higher in ME7+LPS than in all other groups (*P* < 0.05 vs. ME7+saline and *P* < 0.001 vs. NBH+LPS), (Fig. 2c).

The major novel transcripts further elevated in the current study were SRA2 (Fig. 2d), MMP3 (Fig. 2e) and uPAR (Fig. 2f). Peripheral challenge with LPS produced increases in the expression levels of SRA2 in both NBH- and ME7 animals with respect to controls (Fig. 2d). ME7+LPS mice showed a robust increase in expression with respect to all other groups. A one-way ANOVA followed by Bonferroni's post-hoc test confirmed that ME7+LPS induced higher transcription of SRA2 mRNA than either ME7+saline or NBH+LPS (*P* < 0.001). With respect to MMP3 mRNA, ME7+LPS induced these transcripts more robustly than ME7+saline (*P* < 0.05) or NBH+LPS (*P* < 0.001). Conversely, uPAR was significantly increased by LPS in both ME7- and NBH- animals (*P* < 0.001 in both cases), but these were not different to each other (*P* > 0.05) despite ME7-associated levels in ME7 animals being significantly higher than NBH-animal levels (*P* < 0.05). Nonetheless it is notable that systemic LPS induce a marked increase in CNS uPAR transcription, irrespective of its prior inflammatory status.

### Systemic Challenge with LPS Decreases the Expression of Some Phagocytosis-Related Genes

**Fig 3:**
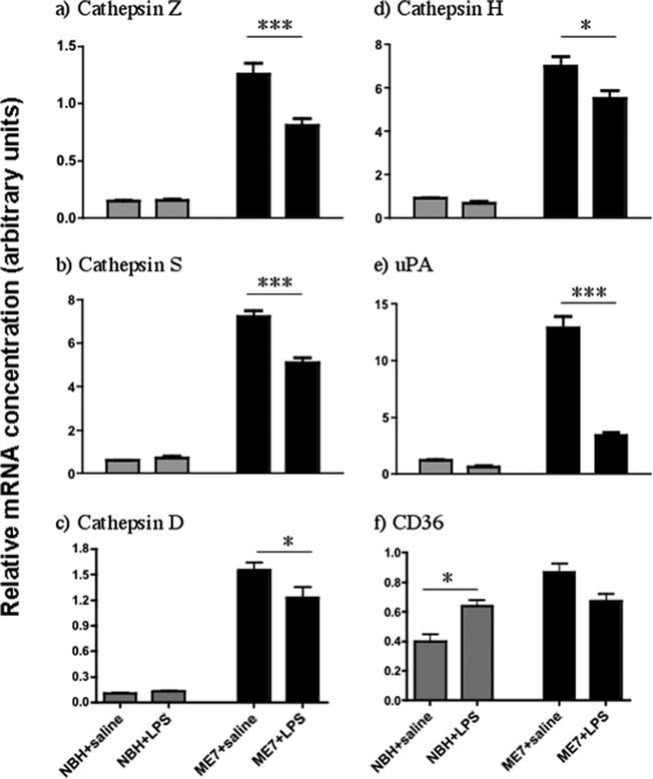
Suppression of inflammatory genes by LPS. Relative mRNA expression for (a) Cathepsin Z, (b) Cathepsin S, (c) Cathepsin D, (d) Cathepsin H, (e) uPA, and (f) CD36 were assessed three hours post-LPS (500 μg kg^−1^). Significant differences after analysis by one-way ANOVA followed by Bonferroni's post-hoc comparisons are shown as *P < 0.05 and ***P < 0.001. *n* = 3 for NBH+saline and *n* = 5 for all other groups.

Relative mRNA expression for Cathepsin Z, S, D, and H were all decreased 3 h after treatment of NBH- and ME7 animals with LPS. (Fig. 3a–d respectively). All four genes followed the same pattern of expression with robust expression in ME7+saline compared with NBH+saline, a minimal effect of LPS in NBH animals and a decrease in mRNA expression upon LPS challenge of ME7 animals. Analysis by one-way ANOVA followed by pairwise comparisons by Bonferroni's post hoc test revealed that LPS significantly lowered transcript levels compared with ME7+saline for cathepsin Z (*P* < 0.001), cathepsin S (*P* < 0.001), cathepsin D (*P* < 0.05) and cathepsin H (*P* < 0.05) while LPS has no effect in NBH animals (*P* > 0.05 for all four cathepsins).

ME7+saline showed a marked increase in uPA mRNA with respect to NBH+saline, but surprisingly this increase was almost completely reversed by treatment of ME7 animals with LPS. One-way ANOVA with Bonferroni post-hoc tests showed that LPS induced a statistically significant decrease in uPA mRNA (Fig. 3e, *P* < 0.001) while having no significant effect in NBH animals (*P* > 0.05). Conversely, LPS has opposite, though mild, effects on the scavenger receptor CD36 in NBH- and ME7 animals: LPS induced CD36 mRNA significantly in NBH animals (*P* < 0.05) but decreased CD36 mRNA in ME7 animals, albeit nonsignificantly (*P* > 0.05).

### Effect of Systemic Challenge with LPS on PrP^Sc^ Clearance

**Fig 4:**
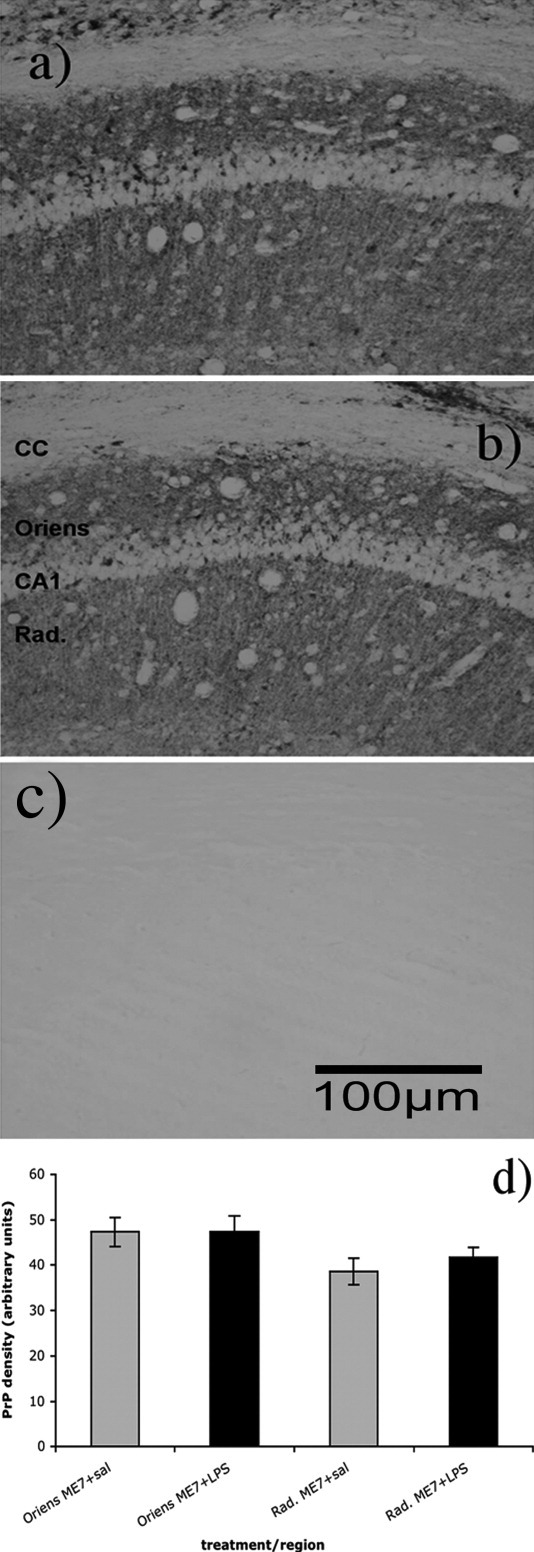
Effect of systemic LPS on CNS PrP^Sc^ levels. PrP^Sc^ deposition was assessed by density of 6D11 staining in the hippocampal strata radiatum (Rad) and oriens in ME7 animals 72 h after injection (i.p.) with sterile saline (grey bars) or LPS 500 μg kg^−1^ (black bars). (a) ME7+saline (b) ME7+LPS (c) NBH. (d) Density calculations for these two layers in ME7+saline and ME7+LPS groups, corrected for density in the corpus callosum (cc). *n* = 3 in all groups. Scale bar = 100 μm.

We examined the density of PrP^Sc^ deposition in ME7 animals injected i.p. with sterile saline or LPS 500 μg kg^−1^. Labeling with the 6D11 antibody after proteinase K pretreatment showed very marked deposition throughout the hippocampus of ME7 animals (Fig. 4a,b) compared with NBH animals (Fig. 4c). However, this density of labeling was not significantly different in ME7+saline and ME7+LPS animals in either the stratum oriens (*P* = 0.99) or the stratum radiatum (*P* = 0.37) when assessed by students *t* tests (Fig. 4d).

### Effect of Intracerebral Challenge with LPS on PrP^Sc^ Clearance

**Fig 5:**
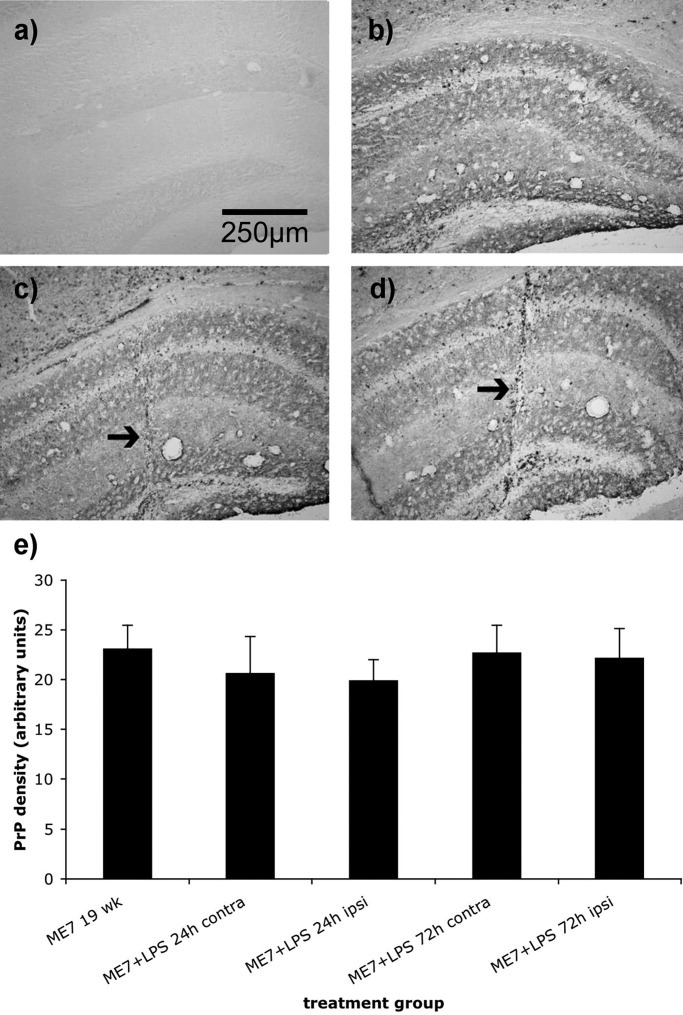
Effect of centrally administered LPS on CNS PrP^Sc^ levels. PrP^Sc^ deposition was assessed by density of 6D11 staining in the hippocampal strata radiatum (Rad) in ME7 animals 24 or 72 h after injection (i.c.) with LPS 0.5 μg. (a) NBH+LPS (b) ME7 sham (c) ME7+LPS 24 h (d) ME7+LPS 72h. (e) Density calculations for the stratum radiatum proximal to the injection tract in all ME7 groups compared with the contralateral side and to sham operated ME7 animals. All density measures were corrected for density in the corpus callosum as an internal standard. *n* = 3 in all groups. Scale bar = 250 μm. The injection tract through which i.c. LPS was delivered is marked in c and d by arrows.

Immunohistochemical labeling with the anti-PrP antibody 6D11 revealed deposited PrP^Sc^ after destruction of the normal cellular form, PrP^c^, of the protein. Thus, NBH animals showed no 6D11-positive staining (Fig. 5a) while all ME7 animals showed very marked deposition of PrP^Sc^ throughout the hippocampus (Fig. 5b–d). There was no difference in 6D11 labeling between different ME7-animal groups, PrP^Sc^ staining was equivalent in ME7 animals 24 or 72 h post-LPS (Fig. 5c,d) to levels in sham-operated ME7 animals (Fig. 5b). The tract of the micro-capillary through which LPS was delivered is visible in Fig. 5c,d. Despite the proximity to the area of acute inflammatory exacerbation, there was no evidence of clearance of PrP^Sc^ in this area. Density analysis of PrP^Sc^ labeling in the stratum radiatum directly adjacent to the injection tract compared with that on the contralateral side and to that in SHAM operated ME7 animals showed no significant clearance in the region of LPS stimulation (Fig. 5e). There were no significant differences between any of the groups by one-way ANOVA (*P* = 0.87). Thus focal challenge with LPS did not produce any focal clearance of this amyloid protein.

### Microglial Phenotypic Switching After Intracerebral LPS

**Fig 6:**
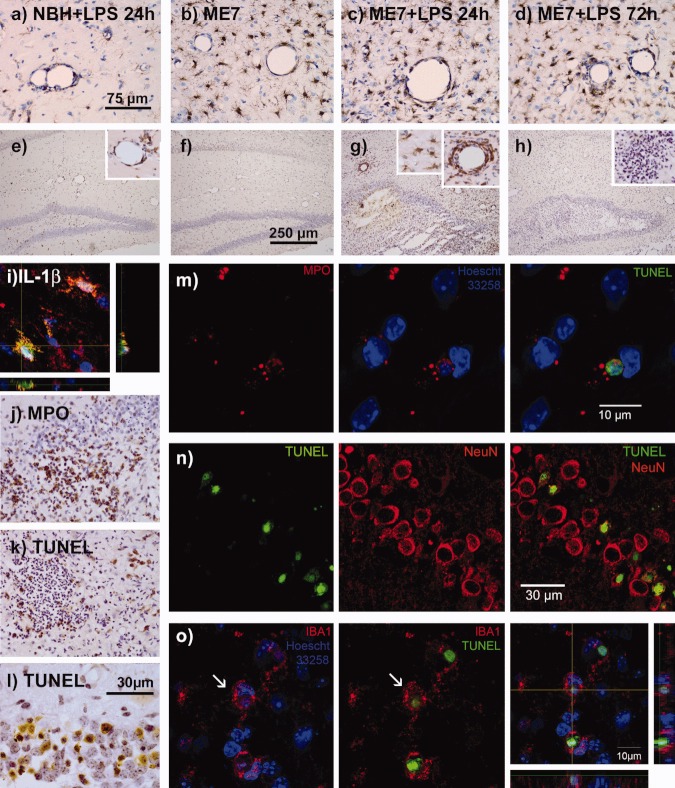
Inflammatory exacerbation, neuronal and neutrophil apoptosis and phagocytosis of apoptotic cells. Microglial distribution and IL-1β expression were assessed by immunohistochemistry in NBH+LPS (24-h post-LPS), ME7 (without LPS), and ME7+LPS 24 and 72 h after intracerebral challenge with 0.5 μg LPS. IBA-1 labeling revealed increased microglial numbers and condensed morphology in ME7 (b–d) groups compared with NBH (a). Anti-IL-1β antibody reveals that IL-1β is absent in ME7 animals (f), is induced by LPS (24 h) in both microglia and infiltrating cells in ME7 animals (g, insets) and to a much lesser degree in NBH+LPS animals (e) but is once again absent by 72 h (h, inset). (i) Double labeling of IL-1β and IBA-1 shows IL-1β-expressing microglia at 24-h post-LPS. (j) Immunohistochemistry for MPO reveals marked neutrophil infiltration at 24 h in ME7 animals post-LPS. All subsequent images are from ME7+LPS at 72 h. TUNEL labeling reveals many apoptotic neutrophils (k) and neurons (l) at 72 h after LPS injection and these are confirmed as neutrophils by double labeling for MPO and TUNEL (m) or as neurons by double labeling for NeuN and TUNEL (n). (o) Double labeling for IBA-1 and TUNEL shows phagocytosis of apoptotic cells (i.e., complete engulfment of apoptotic nucleus by IBA-1-positive cell (arrows). Hoescht 33258 counterstain is shown in blue. Scale bar = 75 μm in a–d, j, and l, 250 μm in e–h, 30 μm in l and n and 10 μm in m and o.

It was predicted that this focal stimulation with LPS would also produce marked escalation of local inflammation. Microglial distribution was examined in NBH- and ME7 animals and also assessed following secondary stimulation with LPS using immunohistochemistry for IBA-1. NBH+LPS (24-h post-LPS) showed sparsely distributed microglial cells with ramified morphology (Fig. 6a) while ME7 animals showed considerably higher density of IBA-1-positive cells and these were more condensed in appearance, though still displaying processes (Fig. 6b). There were no obvious changes in microglial morphology or distribution 24-h post-LPS (0.5 μg i.c.; Fig. 6c). At 72-h post-LPS many microglia showed distribution and morphologies that appeared unchanged from those evident in ME7 animals without LPS and those 24 h after LPS (0.5 μg i.c.; Fig. 6d). However there were also many IBA-1-positive cells with a more condensed morphology, particularly in areas of marked cell infiltration. This is discussed with respect to apoptotic cell death below.

Despite their consistent morphological appearance, many microglial cells were IL-1β-positive in ME7 animals 24-h post challenge with LPS (Fig. 6g). IL-1β expression was accounted for by *de novo* synthesis in previously primed microglia and by infiltrating cells (insets Fig. 6e,g). Microglial cells were unambiguously demonstrated to express IL-1β using double-immunohistochemistry (Fig. 6i). IL-1β-positive cells were very few in number in NBH animals 24-h post-LPS (Fig. 6e) and completely absent in the brains of ME7 sham controls (Fig. 6f). This expression of IL-1β had completely resolved by 72 h (Fig. 6h).

LPS in ME7 animals induced very considerable cellular infiltration, demonstrating a significant exacerbation of local inflammation (Fig. 6g,h). Further examination of the site of focal exacerbation of inflammation showed that the post-LPS cellular infiltrate was largely composed of neutrophils as illustrated by their marked expression of myeloperoxidase (Fig. 6j). At 72 h after LPS injection, many of these neutrophils become apoptotic. These apoptotic cells can be demonstrated by TUNEL staining and nuclear condensation and can tentatively be identified as neutrophils (6k) and neurons (6l) by their localization in the tissue. Many neurons of the CA1, CA3, and dentate gyrus became apoptotic by 72-h post-LPS (Fig. 6l). Using double labeling with TUNEL and NeuN or MPO we have shown that the apoptotic cells comprise both neutrophils (6m) and neurons (6n). IBA-1 labeling reveals microglia with condensed morphology, lacking in obvious ramifications, persisting in the vicinity of these apoptotic cells. Double labeling of TUNEL and IBA-1 demonstrates that these apoptotic cells are in the process of being phagocytosed by local IBA-1-positive microglia (Fig. 6o). That is to say, TUNEL positive nuclei are completely engulfed by IBA-1-positive cells (Fig. 6o middle panel). When only IBA-1 and the Hoescht counterstain are viewed these cells can be seen to contain two nuclei: the microglial nucleus and the shrunken apoptotic nucleus (Fig. 6o left panel). Despite the proximity of microglia and apoptotic cells, and indeed the active phagocytosis of these apoptotic cells the microglia/macrophages of the ME7-animal's brain 72-h post-LPS remain negative for IL-1β (Fig. 6h, inset). These data indicate that microglia proximal to apoptotic cells and engaged in phagocytosis of these cells, remain in an antiinflammatory state, at least with respect to IL-1β synthesis, and support the idea that phagocytic state does not necessarily define inflammatory mediator production.

## DISCUSSION

The aims of this study were to investigate the ability of microglia in animals with prion disease to demonstrate phagocytic capacity and to examine whether subsequent systemic or central LPS challenges alter the clearance of disease-associated deposited PrP^Sc^. We have shown that there is very significant transcriptional upregulation of a wide array of phagocytic and proteolytic-related transcripts in animals with prion disease (ME7 animals) compared with NBH-animal controls. Consistent with this, microglia in ME7 animals were effective in engulfing intra-hippocampally-injected 6 μm inert latex beads while these were largely ignored in the hippocampus of NBH animals. Systemic inflammation induced significant further activation of inflammatory transcripts with further upregulation of SRA2, MMP3, and uPAR but downregulation of transcripts for uPA, CD36, and the cathepsins Z, S, D, and H. The latter data predicted that increased inflammatory activation would not lead to increased phagocytosis of deposited PrP^Sc^ and indeed neither intraperitoneal nor intracerebral LPS, despite the latter causing marked focal acute exacerbation of inflammation and apoptotic cell death did not result in any focal clearance of deposited PrP^Sc^. However, microglial cells were engaged in phagocytosis of apoptotic cells, and maintained an antiinflammatory phenotype during this activity.

### Microglial Activation State During Prion-Associated Neurodegeneration

We (Perry et al., 2002), and others (Baker et al., 1999; Brown et al., 2003), have described the microglial phenotype during prion disease progression. These cells show increased numbers, altered morphology and increased expression of many cell surface markers indicative of activation (Betmouni et al., 1996; Williams et al., 1994). However, the inflammatory mediator profile shows robust expression of TGFβ1 and PGE2 and very low levels of the classical proinflammatory cytokines (Cunningham et al., 2002; Walsh et al., 2000; Walsh et al., 2001). This profile resembles that of peripheral macrophages engaged in phagocytosis of apoptotic neutrophils (Fadok et al. 1998; Savill et al. 2002) and cultured microglial cells engaged in phagocytosis of PC12 cells or of phosphatidyl serine bearing liposomes (a mimic of apoptotic cells), (De Simone et al., 2003, 2004). Although degenerating neurons do appear to be removed, there is evidence that early degenerating synaptic boutons are not stripped by microglia (Siskova et al., 2009). Collectively these observations suggest tight regulation of the microglial phenotype when exposed to neuronal debris arising from chronic neurodegeneration.

Given the persistence of high levels of extracellular amyloid (PrP^Sc^) in these models it seemed reasonable to ask whether the muted inflammatory phenotype described above was responsible for the failure to remove this extracellular amyloid. There are also indications in the literature that astrocytes may display phagocytic capacity (Cahoy et al., 2008) but the persistence of PrP^Sc^ suggests a failure also of these cells to clear this amyloid. A linear model of microglial activation, in which the phagocytic state is the most proinflammatory, has been widely accepted (Raivich et al., 1999; Streit et al., 1999). Here we have observed that, across a wide range of phagocytic and proteolytic markers, all of which can be expressed by microglia and many of which are myeloid lineage restricted, there were very marked up-regulations of transcripts. Since these markers are not all microglial specific, and since their expression does not prove phagocytic activity, we also directly examined microglial phagocytosis of latex beads. Microglial phagocytosis of these inert beads was significantly greater in ME7 animals than in NBH-animal controls. Thus, microglial phagocytic capacity is evident during disease in the absence of marked upregulation of proinflammatory cytokines. However, despite this upregulation of phagocytic machinery, PrP^Sc^ persists and accumulates in the tissue. In other amyloidoses macrophages are rather poor at removing amyloid by phagocytosis, extracellular proteolysis or other means and it has been shown that acute phase proteins such as serum amyloid protein and other pentraxins can coat amyloid proteins and prevent its phagocytosis (Botto et al., 1997; Tennent et al., 1995). Previous studies have also shown that peptides from the prion protein, when coated onto beads, can prevent their phagocytosis, suggesting that the prion protein itself is an inhibitor of phagocytosis (Ciesielski-Treska et al., 2004). The current study shows that the failure to clear PrP^Sc^ is not a failure of the phagocytosis apparatus.

### Acute Inflammatory Activation and Clearance of Amyloid

Another possible explanation for the failure to clear PrP^Sc^, was a failure to induce appropriate proinflammatory pathways which might then lead to proteolyis and/or phagocytosis. We have previously shown that both systemic and central LPS challenges can change the secretory profile of microglia that have been primed by prior neurodegeneration and that this occurs without further changes in microglial morphology (Cunningham et al., 2005). The current studies interrogated whether this phenotypic change was sufficient to induce enhanced clearance of PrP^Sc^ proximal to this region of inflammatory stimulation. Several studies have now suggested that clearance of amyloid-β can be enhanced by further inflammatory stimulation in mice and humans (Akiyama and McGeer, 2004; DiCarlo et al., 2001; Herber et al., 2004). However, despite profoundly increased focal inflammation in the hippocampus following intracerebral injection of LPS no clearance of deposited PrP^Sc^ was apparent in our studies. Marked IL-1β synthesis in local microglia was evident at 2 h (Cunningham et al., 2005) and marked neutrophil infiltration was obvious by 24 h. Thus even acute stimulation of resident cells and recruitment of leukocytes does not appear to affect any clearance of PrP^Sc^ amyloid. This is at variance with the recent studies in mouse models of Alzheimer's disease, which suggest that LPS stimulation can affect clearance of β-amyloid. One possible explanation would be that the inflammatory profiles of microglia in the ME7 (prion) model and the APP and APP/PS1 (Alzheimer's) models are divergent. However the cytokine expression profiles in these amyloid precursor protein transgenic models are not markedly different to those profiles observed in the ME7 model: a rather limited age-dependent expression of proinflammatory cytokine transcripts is observed and levels of these transcripts are significantly acutely increased by systemic challenge with LPS (Sly et al., 2001) just as we have observed in ME7 animals (Cunningham et al., 2005).

The studies reporting clearance of Aβ in Alzheimer's mouse models following intra-hippocampal stimulation with LPS hypothesize that this clearance was effected by increased microglial phagocytosis (DiCarlo et al., 2001; Herber et al., 2004) but this has not been demonstrated. Reports that this LPS-induced amyloid clearance are inhibited by dexamethasone, do not directly implicate any particular cell type (Herber et al., 2007). It is perhaps informative that in earlier human post-mortem studies, 24 h after stroke, amyloid fibrils were actually demonstrated inside the lysosomes of infiltrating macrophages (Wisniewski et al., 1991) and this was in contrast to the absence of amyloid fibrils in the lysosomes of resident microglia. The latter data suggest that microglia do not phagocytose amyloid to any significant degree and indeed recent studies have shown that ablation of microglia for several weeks has no impact whatsoever on amyloid levels (Grathwohl et al., 2009). There are now a number of studies showing that infiltrating macrophages are targeted to amyloid plaques (Grathwohl et al., 2009; Simard et al., 2006) but the efficiency of this process is debated. More recent studies suggest that only small numbers of plaques are targeted by infiltrating macrophages and clearance of these plaques is by no means complete (Jucker and Heppner, 2008). Thus, whether microglia ever efficiently clear β-amyloid *in vivo* remains unclear, but it is relatively clear that infiltrating macrophages are certainly more efficient in this regard. It is striking that the authors of the prior mouse amyloid studies do not report any cellular infiltration of neutrophils or macrophages despite using intracerebral LPS doses that were several fold higher (4 or 10 μg LPS i.c.) than those used in the current studies (0.5 μg). This may be because amyloid levels were not investigated until 3 or 7 days after the central LPS challenge and considerable cell infiltrations may have resolved by that time. Alternatively, cell infiltration may simply not have been investigated in those prior studies. One more recent study reports augmented clearance of amyloid-β after intra-hippocampal injection of 4 μg LPS, and associates this with increased infiltration of bone marrow-derived macrophages, although phagocytosis by these cells is not directly demonstrated (Malm et al., 2008).

Irrespective of the amyloid species in question, it is clear that any notion of improving clearance of amyloid whether through the further activation of microglia, hypothesized as a potentially beneficial strategy (DiCarlo et al., 2001), or by stimulation of further cell infiltration, must take account of the clear potential negative consequences for such stimulation. Such consequences are likely to include neuronal death and considerable leukocyte infiltration and tissue damage as observed here. Recent postmortem reports of amyloid clearance in patients receiving active vaccination with Aβ have been tempered by the occurrence of terminal dementia despite effective amyloid clearance (Holmes etal., 2008) and by the occurrence of meningoencephalitis in a small subset of patients (Nicoll et al., 2003). Our own studies suggest that even systemic inflammation superimposed upon existing neurodegenerative disease actually exacerbates neurodegeneration and is associated with accelerated disease progression both in animal models and in an Alzheimer's disease cohort (Cunningham et al., 2005, 2009a; Holmes et al., 2009).

### Phagocytic State and Degree of Activation are not Positively Correlated

Consistent with our prior reports in ME7-diseased brain (Perry et al., 2002; Walsh et al., 2001), immunohistochemistry for IL-1β revealed that microglia showing morphological evidence of activation in the diseased brain were not producing the proinflammatory cytokine IL-1β. However, upon intra-cerebral LPS stimulation in the hippocampus marked IL-1β expression was evident at 24 h in the absence of further morphological change. These data are consistent with a phenotypic switch occurring in previously primed microglia. The marked increase in proinflammatory cytokine expression in microglia of the degenerating brain when further challenged with LPS is not associated with increased clearance of amyloid. The observed cytokine expression profile and its consequences for cell infiltration are descriptive of a pro-inflammatory phenotype, and that this does not correlate with increased phagocytosis argues strongly that the linear model of microglial activation (Streit et al., 1999) cannot be universally applied. Furthermore, the appearance of many apoptotic cells in the hippocampus of ME7 animals 3 days post-LPS, afforded us the opportunity to examine the response of local microglia to marked apoptosis in their immediate vicinity. These apoptotic cells comprise dying neutrophils that have recently infiltrated the brain as well as local dying neurons. The apoptosis of infiltrating cells is a necessary stage in resolution of inflammation. In this context it is significant that the microglia that are in the vicinity of these large numbers of apoptosing cells, and in some cases that have engulfed apoptotic cells, remain IL-1β negative. This finding provides *in vivo* evidence that microglia engaged in phagocytosis of apoptotic cells do not express IL-1β and is consistent with the idea, previously shown *in vitro* (Takahashi et al., 2005), that phagocytosis of apoptotic cells by microglia induces an antiinflammatory phenotype.

### A Post-Apoptotic Landscape?

Given the increased inflammation induced by LPS it is of interest that a number of the early transcriptional changes in our studies are for gene products associated with apoptosis. PTX3 is thought to be involved in opsonization of pathogens and apoptotic debris for phagocytosis by cells expressing the Fcγ receptor (Rovere et al., 2000). IgG is increased in the brain of ME7 animals and the activating Fcγ receptors are also increased in microglia during neurodegeneration in this model and further increased by systemic LPS (Lunnon et al., submitted).

SRA2, is a myeloid-lineage specific phagocytic pattern recognition receptor (Platt et al., 1998) and here shows increased mRNA expression in ME7 animals followed by a further rise after LPS stimulation. SRA2 was also found to be elevated in an optic nerve crush model of Wallerian degeneration (Palin et al., 2008) and to be further elevated upon systemic LPS challenge, correlating with enhanced phagocytosis of axonal debris. These data are consistent with the idea that it may be important in the response to neuronal debris. Conversely, the class B scavenger receptor, CD36, which is the scavenger receptor most consistently associated with phagocytosis of β-amyloid (Bamberger et al., 2003; El Khoury et al., 2003) was only slightly increased during prion disease and was down-regulated upon LPS stimulation.

Among the MMPs examined, only MMP3 was elevated in ME7 animals and further increased by systemic LPS. MMP3 is known to be upregulated by LPS (McClain et al., 2009; Mun-Bryce et al., 2002) and by IL-1β (Noh et al., 2009) and its expression is widely described in both neurons and microglia. Neuronal MMP3 has been shown to be an initiator of apoptosis and its subsequent release to be a mechanism of signalling apoptosis to proximal microglia (Choi et al., 2008; Kim et al., 2005) while other studies have implicated microglial MMP3 in post-ischemic neurodegeneration (Walker and Rosenberg, 2009).

uPAR was robustly increased in both NBH- and ME7 animals after intraperitoneal LPS treatment. uPAR is a glycosylphosphatidyl inositol (GPI)-linked cell surface protein and its expression has been reported on microglia activated by diverse stimuli (Cunningham et al., 2009b). Recent studies have suggested that uPAR plays a role in promotion of efferocytosis (phagocytosis of apoptotic cells), (D'Mello et al., 2009). When bound to its receptor uPA functions as a membrane-anchored protease, cleaving plasminogen to form active plasmin. The significance of its marked upregulation during prion disease and obvious downregulation upon subsequent LPS stimulation is currently under investigation.

TREM2 is a myeloid-specific membrane bound receptor expressed by microglia and up-regulated surrounding deposited amyloid plaques (Frank et al., 2008). Stimulation of TREM2 leads to microglial cytoskeletal reorganization forming active phagocytic cells. It is thought that TREM2 acts to maintain an antiinflammatory environment in the diseased brain (Takahashi et al., 2005). Consistent with this LPS has been reported to down-regulate TREM2 in resting microglia (Neumann and Takahashi, 2007). However, in the current study LPS did not suppress transcription of TREM2 and thus it may continue to maintain an anti-inflammatory influence in the brain. This is consistent with its reported role as a key molecule in the non-inflammatory phagocytosis of apoptotic cells.

## CONCLUSION

In this study we have shown that a broad panel of genes involved in phagocytosis and extracellular proteolysis are upregulated in the prion-diseased CNS and the microglia have the capacity to engage in phagocytosis of large inert latex beads. Despite this phagocytic profile deposition of fibrils of PrP^Sc^ continues unabated. Furthermore, secondary challenge with LPS in ME7 animals, either systemically or intracerebrally does not stimulate more efficient clearance of PrP^sc^ although theses challenges do induce marked acute exacerbation of inflammation. Considerable apoptosis occurs after central LPS challenge and microglia engaged in the clearance of these cells adopt a phenotype that is consistent with the non-inflammatory phagocytosis of apoptotic cells. Collectively the data are consistent with the idea that inflammatory state and phagocytic activity are not positively correlated and also suggest that phagocytosis of amyloid and of apoptotic cells may have different consequences for inflammatory phenotype.
