# Supplementary material for: Microglia in the degenerating brain are capable of phagocytosis of beads and of apoptotic cells, but do not efficiently remove PrPSc, even upon LPS stimulation
Source: Glia. 2010 Sep 27;58(16):2017–30. doi: 10.1002/glia.21070 (PMC3498730; doi:10.1002/glia.21070)
Supplement: Supplementary file 1 [file glia0058-2017-sd1.doc]

###### Supporting Information Table 1. Taqman primer and probe sequences.

| Target | Accession  number | Oligo | Sequence | Amplicon  size (bp) |
| --- | --- | --- | --- | --- |
| IL-1β | M15131 | Forward primer | 5’-GCACACCCACCCTGCA-3’ | 69 |
|  |  | Reverse primer | 5’-ACCGCTTTTCCATCTTCTTCTT-3’ |  |
|  |  | Probe | 5’-TGGAGAGTCTGGATCCCAAGCAATACCC-3’ |  |
| COX-2 | NW_000157 | Forward primer | 5’-GAGTGGTAGCCAGCAAAGCC-3’ | 81 |
|  |  | Reverse primer | 5’- TTTAATTGGGAACCCTTCTTTGTT-3’ |  |
|  |  | Probe | 5’-AGCAACAAAAGCGTTCTACAAAGGAACTAACCA-3’ |  |
| iNOS | U43428 | Forward primer | 5’-CAGCTGGGCTGTACAAACCTT-3’ | 95 |
|  |  | Reverse primer | 5’-CATTGGAAGTGAAGCGTTTCG-3’ |  |
|  |  | Probe | 5’-CGGGCAGCCTGTGAGACCTTTGA-3’ |  |
| PTX3 | X83601 | Forward primer | 5’-ACAACGAAATAGACAATGGACTTCAT-3’ | 62 |
|  |  | Reverse primer | 5’-CTGGCGGCAGTCGCA-3’ |  |
|  |  | Probe | 5’-CCACCGAGGACCCCACGCC-3’ |  |
| MMP3 | NM_010809.1 | Forward primer | 5’-GGAAATCAGTTCTGGGCTATACGA-3’ | 112 |
|  |  | Reverse primer | 5’-TAGAAATGGCAGCATCGATCTTC-3’ |  |
|  |  | Probe | 5’-AGGTTATCCTAAAAGCATTCACACCCTGGGTCT-3’ |  |
| MMP9 | NM_013599 | Forward primer | 5’-CGAACTTCGACACTGACAAGAAGT-3’ | 114 |
|  |  | Reverse primer | 5’-GCACGCTGGAATGATCTAAGC-3’ |  |
|  |  | Probe | 5’-TCTGTCCAGACCAAGGGTACAGCCTGTTC-3’ |  |
| MMP12 | NM_008605.3 | Forward primer | 5’-GAAACCCCCATCCTTGACAA-3’ | 129 |
|  |  | Reverse primer | 5’- TTCCACCAGAAGAACCAGTCTTTAA-3’ |  |
|  |  | Probe | 5’-AGTCCACCATCAACTTTCTGTCACCAAAGC-3’ |  |
| Cat Z | NM_022325.4 | Forward primer | 5’ – CCAAGGACCAAGACTGTGACAA – 3’ | 118 |
|  |  | Reverse primer | 5’ – CCGGACAGGGAACCGTAAT -3’ |  |
| Cat H | NM_007801.2 | Forward primer | 5’ – ACCGTGAACGCCATAGAAAAGTT -3’ | 147 |
|  |  | Reverse primer | 5’ – TGTGTGGTTCCTCTGGTTGTG -3’ |  |
| Cat S | NM_021281 | Forward primer | 5’- GCCACTAAAGGGCCTGTCTCT –3’ | 80 |
|  |  | Reverse primer | 5’- TCGTCATAGACACCGCTTTTGT –3’ |  |
| Cat D | NM_009983.2 | Forward primer | 5’-GGC GTC TTG CTG CTC ATT CT-3’ | 90 |
|  |  | Reverse primer | 5’-CCG ACG GAT AGA TGT GAA CTT G-3’ |  |
| SRA2 | L04274 | Forward primer | 5’-GTTCCTGTGTCATGCCATGC-3’ | 76 |
|  |  | Reverse primer | 5’-ATGTCAATGGAGGCCCCA-3’ |  |
| CD36 | NM_016741.1 | Forward primer | TGCGCTCGGCGTTGT | 78 |
|  |  | Reverse primer | GGGTCTATGCGGACATTCTTG |  |
|  |  |  |  |  |
| CD68 | NM_009853 | Forward primer | 5’-CAAGGTCCAGGGAGGTTGTG-3’ | 75 |
|  |  | Reverse primer | 5’-CCAAAGGTAAGCTGTCCATAAGGA-3’ |  |
| RAGE | NM_007425 | Forward primer | 5’-GCAAAGAAACACTCGTGAAGGA-3’ | 126 |
|  |  | Reverse primer | 5’-TGAAACTGCAGGAGAAGGTAGGAT-3’ |  |
| TREM2 | NM_031254 | Forward primer | 5’- TGTGGTCAGAGGGCTGGACT-3’ | 68 |
|  |  | Reverse primer | 5’- CTCCGGGTCCAGTGAGGA-3’ |  |
|  |  | Probe | 5’- CCAAGATGCTGGGCACCAACTTCAG-3’ |  |
| CD200R | BC052682 | Forward primer | 5’- AGGAGGATGAAATGCAGCCTTA-3’ | 80 |
|  |  | Reverse primer | 5’-TGCCTCCACCTTAGTCACAGTATC-3’ |  |
| PBZR | NM_009775.4 | Forward primer | 5’-GGAAGCCACCAGGTAGGTTAGG-3’ | 76 |
|  |  | Reverse primer | 5’-GCAGTGCAGAAAGGCAGGTAT-3’ |  |
| uPA | NM_008873.2 | Forward primer | 5’-GAAACCCTACAATGCCCACAGA-3’ | 127 |
|  |  | Reverse primer | 5’-GACAAACTGCCTTAGGCCAATC-3’ |  |
|  |  | Probe | 5’-CACAATTACTGCAGGAACCCTGACAAC-3’ |  |
| uPAR | NM_011113.3 | Forward primer | 5’-TGCAATGCCGCTATCCTACA-3’ | 116 |
|  |  | Reverse primer | 5’-TGGGCATCCGGGAAGACT-3’ |  |
|  |  | Probe | 5’-CCCTCCAGAGCACAGAAAGGAGCTTGAA-3’ |  |
| tPA | J03520.1 | Forward primer | 5’-GGCCTGGCACGACACAAT-3’ | 66 |
|  |  | Reverse primer | 5’-CATCACATGGCACCAAGGTC-3’ |  |
|  |  | Probe | 5’-ATTGTCGGAATCCAGATGGTGATGCC-3’ |  |
| p22phox | NM_007806 | Forward primer | 5’-CGTCTGGCCTGATTCTCATCA-3’ | 79 |
|  |  | Reverse primer | 5’-GAGTAGGCGCCGAAATACCA-3’ |  |
| p91phox | NM_007807 | Forward primer | 5’-CAGGAACCTCACTTTCCATAAGATG-3’ | 113 |
|  |  | Reverse primer | 5’-TCCCGACTCTGGCATTCAC-3’ |  |
|  |  | Probe | 5’-CACACCGCCATCCACACAATTGC-3’ |  |
| MPO | NM_010824.1 | Forward primer | 5’-TGCTGGAAGGTGGCATTGA-3’ | 88 |
|  |  | Reverse primer | 5’-TCATCCACCACAATTTGATTCTG-3’ |  |

Where probe sequences are not included, SYBR green has been used in their place
